# Indications and Outcome of Patients who had Re-Laparotomy: Two Years' Experience from a Teaching Hospital in a Developing Nation

**DOI:** 10.4314/ejhs.v30i5.13

**Published:** 2020-09

**Authors:** Kirubel Abebe, Biniyam Geremew, Befekadu Lemmu, Engida Abebe

**Affiliations:** 1 Department of Surgery, St. Paul's Hospital Millennium Medical College, Addis Ababa, Ethiopia; 2 General Surgeon, Tercha General Hospital, SNNP, Ethiopia

**Keywords:** Re-laparotomy, indications, morbidity, mortality

## Abstract

**Background:**

Complications from abdominal surgery may necessitate a second or more surgeries, re-laparotomy. It is associated with significant morbidity and mortality. Data on relaparotomy from the developing nations is limited. This study aims to assess the indications and outcome of patients who had relaparotomy

**Methods:**

A retrospective review of medical records of all patients who underwent Re-laparotomy at St. Paul's Hospital Millennium Medical College from January 2016 to December 2017 was done.

**Result:**

Of 2146 laparotomies, 6.9% (149) needed re-laparotomy and 129 patients were analyzed. Most (123,95.3%) had on-demand re-laparotomy. Patients operated on emergency made 70.5% (91) of the cases making the ratio of emergency to elective surgery 2.4:1. The three most common surgeries that needed re-laparotomy were, Perforated appendicitis (35,27.1%), bowel obstructions (28,21.7%) , and trauma (20,13.4%). The most common indications for relaparotomy were intra-abdominal abscess (57,44.23%), wound dehiscence (17,13.2%) and anastomotic leak (15 ,11.6%). Surgical site infection (128,100%) and malnutrition (58,45%) were the leading complications. The overall mortality rate was 12.8 % (19). There was no statically significant difference in mortality rate between on-demand and planned re-laparotomy (P=0.388), urgency of the primary surgery (P=0.891) and the number of relaparotomy (p=0.629). Re-laparotomy for anastomotic leak (p=0.001) and patients above fifty years of age (P=0.015) had significant associations with mortality.

**Conclusion:**

Intra-abdominal abscess collection, wound dehiscence and anastomotic leak were the most common indications of re-laparotomies. Age above fifty years and anastomotic leaks were significantly associated with mortality.

## Introduction

Laparotomy is a surgical incision into the abdominal cavity for diagnosis or in preparation for major surgery. Re-laparotomy (RL) is a planned or an unplanned re-operation carried out during the postoperative period after laparotomy for reasons related to first operation. RL is done mainly for complications from the primary abdominal surgery and can be associated with significant morbidity and mortality (1–6). There is no general consensus as to which patients should undergo RL. The decision is frequently challenging, especially when one faces critically ill patients with non-specific signs and symptoms of partially treated sepsis. The need to do RL and its timing is also subjective. In general, there are two approaches (strategies) of RL: Planned Relaparotomy (PLR) and On-demand Re-laparotomy (ODRL) (5–18).

ODRL is done when the patient's clinical condition deteriorates or lacks improvements after the first laparotomy or when there are radiologic or laboratory evidence of pus, bile or intestinal contents in drains placed in the abdominal cavity during the first operation. Other indications for ODRL include: medical treatment-resistant hemorrhage, presence of post-operative peritonitis or abscess where percutaneous drainage is not feasible or effective. An ileus resistant to medical treatment or decompression can be also an indication for ODRL (2–4,13–18).

Factors which influence outcomes of patients who underwent RL includes patient's sociodemographic characteristics, the indication for the first operation, the urgency of the first operation, the duration between first operation and RL, etc. (2,9,14,16,17). RL done for dehiscence and early obstruction carries minimal risk while RL for bleeding and infection entails moderate risks. The highest rate of mortality is when RL is done for anastomotic leak (2,13–16). The mortality rate is higher in older age groups and multiple RL (1,2,7,15).

Little has been published about this topic in Ethiopia and in Africa. To the best of our knowledge, there is only one published data done by Negussie et al. at Tikur Anbessa Specialized Hospital on pediatrics re-laparotomies (6). Reports by Scriba et al and Ugumba et al are the two relaparotomy studies retrieved during our literature review in Africa (7,8). This study also helps to analyze the magnitude of the problem, to improve the care, to establish preventive strategies, and it can provide a baseline data for further study. On the background of the above reasons, our study aims to determine the indications and outcome of patients who undergone RL.

## Methods and Materials

This was a facility-based cross-sectional study of all patients (age >12 years) who underwent RL at St. Paul's Hospital Millennium Medical College (SPHMMC) from January 2016 to December 2017. SPHMMC is a tertiary referral teaching hospital in Addis Ababa, Ethiopia. The hospital had 10 general surgeons, 3 urologists and 20 general surgery residents during the study period.

The term “Relaparotomy” is defined as a planned or an unplanned re-operation carried out during the hospitalization postoperative period after laparotomy for reasons related to first operation. “Planned Re-laparotomy (PLR)” is defined as RL at regular 48-hour intervals until adequate source control has been achieved, and “On-demand Relaparotomy (ODRL)” is RL for only those with signs of unresolved intra-abdominal conditions or new complications.

All patients (age >12 years) who underwent laparotomy at the hospital were identified, and patients who required at least one RL were studied. Pediatric patients, patients operated on at Gynecology and Obstetrics Department or patients who had their first laparotomy elsewhere were excluded. Patients' medical record numbers (MRNs) were identified from operation theater log books and nursing admission/discharge registration book. Charts of patients who had at least one RL were retrieved from hospital archive room.

An MRN of 149 patients who underwent RL were identified in the study period, and 129(86.6%) patient charts were included for reasons such as incomplete data and missed charts. Data on sociodemographic characteristics, primary indications for the surgery, indications for re-laparotomy, types of post-operative complications and duration of hospital stay were extracted retrospectively from individual charts using a pretested data collection format by trained third year surgical residents.

Data was checked for completeness, accuracy and consistency then coded and entered into SPSS version 20 for analysis. Descriptive statistical techniques were used to characterize the variables and determine outcome of patients after RL. Results were shown using tables, graphs and central tendency statistics. Association between outcome (mortality) and independent variables (sex, age, indication for RL, type of RL, nature of primary surgery and frequency of RL) was done and considered significant when p-value was <0.05. A written ethical clearance letter was obtained from SPHMMC, the Institutional Review Board.

## Results

A total of 2146 patients underwent laparotomy in the two years' period. RL was performed in 149(6.9%). Of these, 129 patients were analyzed. The mean and median age of patients who needed RL was 37.8 years (SD +/-14.7) and 35 years (15 to 68 years). Almost equal number of males (65,50.4%) and females (64,49.6%) had RL. RL was commonly done for patients who had emergency surgeries (91,70.5%) making the ratio of emergency to elective surgery 2.4:1. The majority (123,95.3%) of the patients had ODRL. Timing of decision to do RL ranged from the immediate post-operative day (for hemorrhage) to the 12^th^ post-operative day making the median 5.1 days. The majority (113,87.6%) of the patients underwent RL once, and the average number of RL was 1.31.

The top three emergency surgeries which needed relaparotomy were perforated appendicitis (35,27.1%), bowel obstructions (28,21.7%) and trauma 20(13.4%). Among elective operations of cholelithiasis and gasterointestinal (GI), malignancies top the list, 9.3%(12) each.

The most common indication for RL was intraabdominal abscess (57,44.23%), followed by wound dehiscence (17,13.2%). The highest case fatality rate (CFR) was seen in patients with anastomotic leak (7,46.7%).

The 129 patients had developed one or more post-operative complications after the RL. SSI was seen in all of the patients followed by malnutrition (58,45%) and anemia requiring transfusion (42,32.5%).

Overall mortality rate was 14.7% (19). Most patients (13,68.4%) died due to multiple organ failure (MOF/sepsis), followed by pulmonary embolism in (4,21%) and for rest of the cases (2,10.5%), the causes of death were not documented. Post-operative stay of patients ranged from 9 to 46 days with a median 22 days.

Patients with anastomotic leak as a cause of RL (p=0.001) and age above 50 years (p=0.015) had significant association with mortality (p=0.001). Otherwise, sex, urgency/nature of the primary surgery, type and number of RL had no significant associations with mortality.

## Discussion

The reported rate of re-laparotomy in developed countries ranges from 1% to 5.7% (1,2,11,14–16). On the other hand, studies from Africa found higher rates: 17.2% Ethiopia, 21% South Africa and 18% Congo (6–8). This difference could be due to many factors such as hospital setup, indication for the first surgery, type of surgery and study population (1–3,11,14–16). The lower rate (6.9%) in this study than the African studies reflects difference in study subjects. In both studies, patients referred from other centers for RL were included, and the Congo study involved both adult and pediatric patients. In the South Africa study, trauma cases comprised almost one third of the study population which is 15% in our study due a separate trauma center (7,8). The previous Ethiopian study by Negussie et al was entirely done on pediatric surgical patients and reported a high rate of RL which could be due to the high burden of pediatric surgery as Tikur Anbessa Teaching Hospital serves as a primary referral center for all pediatric surgical patients in the country (6). The availability of interventional radiology (percutaneous drainage) may contribute to the lower rate in developed countries (15,16).

Most studies revealed male predominance (1,2,7,8,14,15,16). In contrast, a study by Wain Mo et al revealed higher RL rate in females (17). Our study and Negussie et al's demonstrated similar rates in both sexes. This may reflect differences in study subjects and disease incidence (2,17). The mean age of patients who had RL in the western countries were higher than African and Indian reports (1,2,4,6–8,14–17). The high life expectancy and disease pattern, more malignant condition in the western world which tend to occur in older age, may contribute to this discrepancy. The South African and Congo studies reported 38 and 34.6 years which is similar with our finding (37.8 years) (7,8).

In our study, most RL were done for emergency surgeries than elective (70.5% Vs 29.5%) which is in line with other studies (2,4,6–7,14–16). Perforated appendicitis, bowel obstructions and trauma were the top surgeries complicated and required RL in this and many other studies (3,6–9,14,17). On the contrary, studies from developed nations demonstrated GI malignancies as the most common index surgery (1,15). This may reflect the difference in disease incidence. Our review of records and the African studies showed the most common index surgery required RL was appendicitis (6–8). The reason could be delayed presentation and inadequate surgical service at the primary hospital. However, this needs further study. As reported by this analysis and others, most patients underwent ODRL (1,2,14–16). The South African study demonstrated higher number of PRL (41%) due to the higher burden of trauma among patients demanding damage control surgery (7). This study also identified the average number of RL being similar with reports of Unalp HR et al (1.46) and Prabhu S et al (1.38) (3,15).

Intra-abdominal abscess/collection, wound dehiscence and anastomotic leak were the leading indications in our and other studies (1,3,6–8,14,15,17). In contrast, studies done by Koirala R et al and Ching SS et al revealed hemorrhage being the commonest indication which is very low in our patients (2,16). This discrepancy can be explained by the complexity and type of the first surgery. In both studies, there is high burden of liver and pancreatic surgery which were usually complicated by bleeding.

Literatures reported that overall mortality rate ranged from 20% to 40% (1–3,13–16,18). Our review (14.7%) and studies of South Africa (14%) and Congo (17.6%) revealed comparable rate each other but lower than the above reported range (7,8). The study subjects in our review were relatively younger and had benign disease which may contribute to the difference. On the other hand, comorbidities and the complexity of the procedures may impose higher mortality in the western studies (1,15). Many authors reported that the mortality rate is mostly affected by the causes/indication of RL (2,14–16). As it is noted in our analysis (P=0.001) and other studies including the Ethiopian study, anastomotic leak caused the highest mortality among the indications of RL (2,6,14–16). In this study, age (>50years) was significantly associated (P=0.015) with mortality which is consistent with reports of other studies (1,2,15). Ching SS et al reported that mortality rate increases with advancing age, rising from 23% in younger patients below 50 to 75% in those over 80 years (2). Other studies done in India and Europe also revealed association between older age and mortality (1,14). The Ethiopian study demonstrated that neonatal age (< 1 month) was found to be an independent risk factor for death following pediatrics re-laparotomy (p=0.013) (7). This study and many other studies did not show any significant difference in mortality which supports the choice of PRL and ODRL one over the other (3,4,10,12,19). Literatures also reported that ODRL did have a substantial reduction in re-laparotomies, healthcare utilization including ICU, and medical costs (4,19). In addition, other authors suggested variables and scoring systems to decide on the choice of RL strategies following secondary peritonitis (5,9). In contrast with our study, reports of Indian and Turkey found that multiple RL was significantly associated with mortality (15,16). This may reflect difference in the index surgery and patients' characteristics. Regarding the cause of death, our study found MOF/sepsis being the commonest reason (63.1%). This is similar with other findings which ranged from 40%–64% (3,6–8,14,15,16).

In summary, perforated appendix was the most common primary surgery which requires relaparotomy. Intra-abdominal abscess collection, wound dehiscence and anastomotic leak were the most common indications of RLs. The mortality rate was lower than other reports and significantly associated with anastomotic leak and age more than fifty years.

Missing patients' medical records and at times incomplete records of patients were the main limitations in this study. However, the study provided data on the magnitude of the problem which helps in improving patients care and as a baseline for further study.

This review suggests frequent follow-up for patients who had RL for anastomotic leak and are older than fifty years of age as it is associated with increased mortality. Since the study is retrospective and done in a single institution, further studies, including predictors of RL following the index surgery, should be conducted for more generalization.

## Figures and Tables

**Table 1:** Indication of the Primary Lapaparotomy among Patients who Required RL at SPHMMC, Addis Ababa, January 2016-December 2017

Indications of the Primary laparotomy	Frequency (n=129)	%
Perforated appendicitis	35	27.1
Bowel obstructions	28	21.7
Trauma	20	13.4
GI malignancy	12	9.3
Symptomatic Cholelithiasis	12	9.3
PUD perforation	6	4.7
Biliary and pancreatic cancer	3	2.3
Ureterolithotomy/pyelolithotomy	3	2.3
Transvesical prostatectomy	3	2.3
Mesenteric ischemia	3	2.3
CBD exploration	2	1.6
Nephrectomy	2	1.6

**Table 2:** Indications of Re-laparotomy and Case Fatality Rate among Patients Who Needed RL at SPHMMC, Addis Ababa, January 2016 to December 2017

Indications RL	Total n (%)	Mortality n (%)	P value
Intra-abdominal abscess collection	57(44.2)	8(14.0)	0.843
Wound dehiscence and Evisceration	17(13.2)	1(5.9)	0.292
Anastomosis leak	15(11.6)	7(46.7)	0.001**
Biliary leak	12(9.3)	1(8.3)	0.519
Stomal complication	7(5.4)	1(14.0)	0.973
Planned re-laparotomy*	6(4.6)	1(16.7)	0.891
Early post op adhesion	5(3.9)	-	-
Hemorrhage	5(3.9)	-	-
Others	5(3.9)	-	-

* 3 mesenteric ischemia & 3 trauma patients; one patient with mesenteric ischemia has died

** significantly associated at p-value <0.005

**Table 3:** Post-operative Complications Seen among Patients Who had RL at SPHMMC, Addis Ababa, January 2016 to December 2017

Complications	Frequency	Percent
SSI	129	100
Malnutrition (low albumin)	58	45.0
Anemia (requiring transfusion)	42	32.5
Urinary tract infection	39	30.2
Pulmonary complication	35	27.1
Deep vein thrombosis	18	14.0
Enterocutaneous fistula	16	12.4
Others *	7	5.4
**Death**	**19**	**14.7**

* Renal failure (4,3.1%), paralytic ileus (2,1.5%) and DIC/Bleeding (1,0.7%)

**Table 4:** Factors Associated with the Outcome of Patients with RL at SPHMMC, Addis Ababa, January 2016 – December 2017

Variables	Outcome			
	Improved	Died	OR for mortality	P value
**Sex**				
Male	56	9	0.87(0.327–2.301)	0.776
Female	54	10	1	
**Age**				
<=50	69	6	1	0.015*
>50	41	13	0.27(0.097–0.78)	
**Nature of surgery**				
Elective	34	4	1	0.388
Emergency	76	15	0.60(0.184–1.930	
**Type of RL**				
PRL	5	1	1	0.891
ODRL	105	18	0.86(0.095–7.770)	
**Frequency of RL**				
One RL	97	16	1	0.629
More than one RL	13	3	0.71(0.183–2.791)	

* significantly associated at p-value <0.05, OR Odds Ratio
